# Elevated Serum Ceruloplasmin Levels Are Associated with Higher Impulsivity in People with Parkinson's Disease

**DOI:** 10.1155/2020/8296203

**Published:** 2020-09-30

**Authors:** Megan C. Bakeberg, Maddeson Riley, Michelle Byrnes, Alexa Jefferson, Souyma Ghosh, Malcom K. Horne, Sarah McGregor, Rick Stell, Sue Walters, Tess Evans, Katherine Roberts, Frank L. Mastaglia, Ryan S. Anderton

**Affiliations:** ^1^Perron Institute for Neurological and Translational Science, Nedlands, Western Australia, Australia; ^2^Centre for Neuromuscular and Neurological Disorders, University of Western Australia, Nedlands, Western Australia, Australia; ^3^School of Health Sciences, University of Notre Dame Australia, Fremantle, Western Australia, Australia; ^4^Florey Institute for Neuroscience and Mental Health, University of Melbourne, Parkville, Victoria 3010, Australia; ^5^Centre for Clinical Neurosciences and Neurological Research, St. Vincent's Hospital Melbourne, Fitzroy, Victoria 3065, Australia; ^6^Institute for Health Research, University of Notre Dame Australia, Fremantle, Western Australia, Australia; ^7^Centre for Molecular Medicine & Innovative Therapeutics, Murdoch University, Perth, Western Australia, Australia

## Abstract

**Background:**

Heightened impulsivity has been reported in a subset of people with Parkinson's disease (PwP) and is considered a risk factor for the development of impulse control disorders (ICDs). However, at present, there are no recognised biochemical markers of heightened impulsivity.

**Objectives:**

To determine if ceruloplasmin, a serum marker involved in the regulation of iron and copper homeostasis, is associated with trait impulsivity in PwP.

**Methods:**

The study measured serum ceruloplasmin and impulsivity using the Barratt Impulsiveness Scale (BIS-11) in an Australian cohort of 214 PwP. Multivariate general linear models (GLMs) were used to identify whether higher serum ceruloplasmin levels (>75th percentile) were significantly predictive of BIS-11 scores.

**Results:**

Serum ceruloplasmin was higher in females with PD (*p* < 0.001) and associated with MDS-UPDRS III, Hoehn and Yahr, and ACE-R scores (*p* < 0.05). When correcting for covariates, higher serum ceruloplasmin concentrations were associated with the 2^nd^ order nonplanning impulsivity and with the 1st order self-control and cognitive complexity impulsivity domains.

**Conclusions:**

Higher serum ceruloplasmin levels are independently associated with heightened nonplanning impulsivity in PwP. Thus, serum ceruloplasmin levels may have clinical utility as a marker for heightened impulsivity in PD.

## 1. Introduction

Impulsive behaviours in people with Parkinson's disease (PD) have attracted increasing recognition as a burdensome neuropsychiatric manifestation of PD. Impulsivity is known to be a multidimensional construct by nature, likely due to the complexity of personality constructs [1]. Previously, it has been demonstrated that PwP exhibits more impulsive behaviours [2, 3], sometimes referred to as heightened impulsivity. PwP have also been found to perform poorly on a range of measures of response inhibition [4, 5], which is an integral element of impulsivity. Identified in a subset of PwP, heightened impulsivity represents a risk factor for a variety of impulse control disorders (ICDs) [2, 6]. A number of ICDs are thought to be a result of dopaminergic therapies primarily administered to alleviate motor deficits of PD, such as dopamine agonists (DAs) [7]. Despite the strong association between impulsivity and ICDs [2, 6], PwP can manifest heightened impulsivity even in the absence of an ICD [8, 9]. These observations represent impulsivity in PwP on a spectrum of severity and introduce a factor of possible deterioration through the disease course. PwP will often display symptoms of mild cognitive and motor disinhibition, ultimately manifesting as a pathologic ICD over time [6].

Whilst several studies have focused on identifying demographic risk factors for the onset of impulsivity and ICDs in PD [7, 10–13], to date none have investigated serum markers as predictive or associated factors of impulsivity in PD. Previous research has suggested a relationship between PD and serum levels of ceruloplasmin, a multicopper ferroxidase enzyme required for cellular efflux of iron from the central nervous system (CNS) [14], but has not investigated the relationship with impulsivity. In a study using magnetic resonance susceptibility-weighted phase imaging, a close correlation was found between nigral iron content and serum ceruloplasmin levels, with PD subjects with reduced serum ceruloplasmin levels having significantly higher iron content than those with normal ceruloplasmin levels and healthy controls [15, 16]. Thus, low ceruloplasmin levels may be associated with increased cerebral iron deposition in PD although it is not clear if this plays a role in aggravating the neurodegenerative process. On the contrary, elevated ceruloplasmin levels have been associated with a number of psychiatric disorders, such as schizophrenia and obsessive-compulsive disorder (OCD), in which impulsive behaviours can also occur [17, 18]. Furthermore, symptoms of psychosis and schizophrenia-like behaviour have also been reported in selected cases with aceruloplasminemia, a condition characterised by a complete absence of functional ceruloplasmin [19]. Although it remains unclear whether there is a causal relationship between levels of ceruloplasmin and psychiatric disturbances, ceruloplasmin dysfunction is thought to disrupt copper homeostasis [20]. This disruption may contribute to dopamine dysregulation, a prominent feature of these psychiatric disorders and impulsive behaviours [16]. It is not known whether ceruloplasmin levels have an effect on progression of cognitive impairment.

In light of the relationship between ceruloplasmin, PD, and psychiatric disorders associated with dopamine dysregulation, the present study aimed to determine whether there was an association between serum ceruloplasmin levels and impulsivity. Impulsivity and impulsive behaviours were assessed in a cohort of PwP using the Barratt Impulsiveness Scale 11 (BIS-11), which has been extensively used and validated in PD [3, 21].

## 2. Methods

### 2.1. Participants

A total of 214 people with idiopathic PD (IPD) were sequentially recruited from three movement disorders clinics across Australia, as part of the Australian Parkinson's Disease Registry (APDR). All were ambulant and independent with activities of daily living, and none were known to have any other neurological or psychiatric disorder. Furthermore, individuals with any active inflammatory conditions were able to be determined by standard blood tests and could be excluded. All participants were examined and cognitively assessed by a movement disorder neurologist prior to inclusion in the study for verification of the diagnosis in accordance with the UK Brain Bank criteria for IPD [22]. The study was approved by the Sir Charles Gairdner Hospital Human Research and Ethics Committee (Approval number 2006/073), and written informed consent was obtained from all participants, in accordance with the National Health and Medical Research Council of Australia guidelines.

### 2.2. Clinical Assessments

Motor symptoms and disease severity were evaluated in the “ON” state using the MDS-Unified Parkinson's Disease Rating Scale (MDS-UPDRS) Part III and Hoehn and Yahr Scale [23]. In addition, each participant was evaluated by a clinical psychologist and completed a suite of neuropsychological assessments. Cognition was assessed using the Addenbrooke's Cognitive Examination Revised (ACE-R), as previously described [13, 23], a sensitive test of cognition that can be efficiently applied in a clinical or research setting [24]. Whether patients were taking levodopa and/or dopamine agonists was noted. The class of PD medication (carbidopa, Madopar, Sinemet, Stalevo, entacapone, selegiline, and rasagiline) and dosage were recorded; all PD medications were then converted to a total levodopa equivalent daily dose (LEDD). Participants taking D2 dopamine agonists (pramipexole, rotigotine, or apomorphine) were recorded and considered as a covariate due to their known association with ICD risk.

### 2.3. Assessment of Impulsivity

The BIS-11 was employed as a validated self-report questionnaire for screening impulsivity [21]. The BIS-11 consists of 30 questions scored on a four-point scale, with each item corresponding to one of the three BIS-11 2nd order domains. Overall BIS-11 scores were calculated as the sum of these 30 scores (yielding a total score of 120), with higher scores indicating greater impulsivity. The sum of 2nd order BIS-11 attentional, motor, and nonplanning domains were used to calculate participants' BIS-11 2nd order domain scores. BIS-11 2nd order attentional scores were scored out of a maximum of 32, and motor and nonplanning scores were both scored out of a maximum of 44.

### 2.4. Blood Collection and Serum Analysis

Fasting blood samples were collected from participants prior to clinical assessments. For blood collection, 10 ml of whole blood was taken by median cubital vein venepuncture and stored in a standard BD EDTA vacutainer® (Becton Dickinson and Company, Franklin Lakes, N.J.). Serum ceruloplasmin concentrations were quantified using standardised immunoturbidimetric determination assays by State Pathology services, all of which are registered with the National Association of Testing Authorities (NATA) for medical testing.

### 2.5. Data Analysis

Collated data were analysed using IBM-SPSS (v. 26, IBM Corporation). A significant nominal *p* value of ≤0.05 was employed for all statistical tests. Variables were described using mean and standard deviation (in brackets, SD) or frequency and percent (in brackets, %), as appropriate. Normality was assessed using the Shapiro–Wilk test, with subsequent clinical characteristics analysed using independent samples *T*-Test, Mann–Whitney U, or chi-square, as appropriate. Ceruloplasmin blood levels were dichotomised into normal (0–75th percentile) and high (>75th percentile) groups. Total, 2nd order, and 1st order BIS-11 scores were used as participant outcome measures. Wherever appropriate, univariate analysis or Mann-Whitney *U*-test was performed to identify differences between ceruloplasmin groups. Cohen's *d* ESs were calculated for the mean differences, with an ES of 0.20 considered small, 0.50 medium, and 0.80 large. Generalised linear models (GLMs) were conducted in order to analyse the relationship between serum ceruloplasmin and impulsivity, with corrected models controlling for confounding variables. Total BIS-11 scores and BIS-11 1st and 2nd order domain scores were considered separate outcome measures. Residual plots were examined for all models, and no violations were noted.

## 3. Results

### 3.1. Serum Ceruloplasmin Levels and Clinical Characteristics of the PD Cohort

Clinical assessments, demographic information, medications, motor, and cognitive function are presented in Table 1. The cohort was predominated by males, with an average age at assessment of 64.4 years and disease duration of 8.9 years. At the time of the assessment, 46.3% of individuals were taking DAs and had an average LEDD of 884.93 mg/day. Overall, the cohort exhibited a mean total motor score (MDS-UPDRS III) of 19.81 (±14.03), an average of 1.69 (±0.96) on the H&Y scale, and a mean ACE-R of 88.55 (±10.57) (Table 1). Serum ceruloplasmin levels ranged from 0.10 g/L to 0.49 g/L; females appeared to show a greater variability of distribution than males. Furthermore, mean levels were significantly higher in females (0.29 ± 0.07) than males (0.24 ± 0.05; *p* < 0.001). For subsequent analysis, levels of ceruloplasmin were dichotomised into normal (0–75th percentile) and high (>75th percentile) groups.

Mean ceruloplasmin level significantly differed between groups (0.23 ± 0.04 vs 0.34 ± 0.05, *p* < 0.001). Females predominated the group with high ceruloplasmin levels, having a higher mean MDS-UPDRS part III score (23.91 ± 13.94 vs 18.35 ± 13.82, *p*=0.004), a higher H&Y score (2.04 ± 0.99 vs 1.57 ± 0.92, *p*=0.002), and a higher ACE-R score (91.02 ± 8.23 vs 87.72 ± 11.14, *p*=0.034) (Table 2). Subjects within the high ceruloplasmin group did not display any significant differences in age, disease duration, LEDD, or DA usage.

### 3.2. Nonplanning Impulsivity Is Associated with High Ceruloplasmin Levels

Analyses of relationships between ceruloplasmin levels and impulsivity consequently included covariates identified in Table 3. Patients with high ceruloplasmin had significantly higher nonplanning-associated 2nd order impulsivity (*p*=0.012) and related 1st order self-control (*p*=0.047) and cognitive complexity (*p*=0.028) items of the BIS-11 (Table 3). In models correcting for covariates, significant differences remained in 2nd order nonplanning (*p*_adj_=0.003), 1st order cognitive complexity (*p*_adj_=0.020), and 1st order self-control (*p*_adj_=0.009). Thus, such differences were independent of gender and other confounding variables. There were no significant associations identified between ceruloplasmin levels and the attentional or motor impulsivity domains (Table 3). Total BIS-11 scores were higher in the high ceruloplasmin group but did not reach statistical significance.

## 4. Discussion

Impulsive behaviours are well-recognised in a subset of PwP and are thought to underlie the development of more pervasive ICDs [3, 25, 26], which may manifest as pathological gambling and hypersexual and other behaviours [2]. While clinical guidelines and scales have been developed for the identification and diagnosis of ICDs [27], subclinical impulsiveness in PwP is much less well-defined and difficult to identify and categorise. Furthermore, the identification of heightened impulsivity relies heavily upon self-reporting, with patients frequently being reluctant to report impulsive behaviours due to embarrassment and often being unaware of their existence. As such, identification of a serum marker would serve as a useful clinical tool in objectively identifying individuals who are at greater risk of developing ICDs, particularly those who are being treated with a DA. Here, we show that high serum ceruloplasmin levels are significantly related elevated nonplanning impulsivity in PwP, suggesting that serum ceruloplasmin may serve as a convenient marker for impulsivity in PD.

Synthesised in the liver, ceruloplasmin is involved in plasma copper transport [18] and is also thought to be a ferroxidase enzyme, metabolising highly toxic ferrous iron into its nontoxic ferric form [14]. Within the CNS, ceruloplasmin is expressed on the surface of astrocytes and is integral in the mobilisation and efflux of iron in normal, healthy states, preventing the accumulation of toxic ferrous iron within the brain [14]. Serum ceruloplasmin has been reported to be reduced in PwP when compared with healthy control subjects, though evidence is somewhat inconsistent. This reduction of ceruloplasmin may reflect impairments in the ferroxidase activity of ceruloplasmin, which is postulated to promote an accumulation of toxic iron within the substantia nigra of PwP, and in turn, accelerate the neurodegenerative process [15, 28–30]. However, other studies have reported that elevated ceruloplasmin is a feature commonly associated with inflammatory conditions and low-grade chronic inflammation, with its synthesis being induced by inflammatory cytokines and lipopolysaccharide [31–34]. Such literature aligns with the increasingly accepted notion that chronic neuroinflammation plays a central role in the pathophysiology of PD [35, 36].

Increased serum ceruloplasmin has been reported in patients with psychiatric conditions, notably schizophrenia and OCD [17–19, 37]. A common presentation within these neuropsychiatric disorders is impulsivity [38, 39]. Several studies have revealed that patients with schizophrenia report higher levels of impulsivity [40], as assessed by the BIS-11 and the stop-signal and delay discounting tasks [38]. Although findings regarding whether impulsivity underlies OCD are discrepant, some studies have reported that OCD patients also score higher on self-report measures of impulsivity, including BIS-11 [41–43]. Therefore, it is not surprising that increased ceruloplasmin was associated with higher impulsivity in the present PD cohort.

When the different domains of impulsivity were analysed for an association with ceruloplasmin levels, GLM analysis revealed a significant relationship between serum ceruloplasmin levels and BIS-11 1st and 2nd order domains pertaining to nonplanning impulsivity. Nonplanning impulsivity refers to a lack of future planning and consideration for long-term consequences associated with alternate choices and is reflected in a bias toward the present orientation [44, 45]. In general, elevated nonplanning impulsivity is associated with blunted reward anticipation and reduced tolerance for delayed rewards (“increased delay discounting”) [44, 46]. Subjects displaying nonplanning impulsivity may favour immediate, often monetary rewards [44], and as such, nonplanning impulsivity has been associated with problem gambling [47]. A similar trend has been displayed in PwP with pathological gambling, who also score higher on measures of nonplanning impulsivity than PwP without pathological gambling [12]. Therefore, serum ceruloplasmin may serve as a specific marker for nonplanning impulsivity in PwP and may aid in the identification of behaviours associated with aberrant reward processing. Importantly, it may therefore flag harmful behaviours such as pathological gambling, which is one of the most pervasive forms of ICD in PwP [7].

Further research is required to elucidate a potential mechanism that may underlie the relationship between ceruloplasmin and impulsivity in PD. However, we hypothesis that ceruloplasmin may interfere with physiological dopamine regulation, in a similar manner as observed in schizophrenic patients, resulting in the generation of a hyperdopaminergic state. Indeed, dopamine dysfunction appears to play a central role in the development of impulsivity in PD [48]. For instance, PET studies in a subset of PwP exhibiting impulsive-compulsive behaviours have shown that there is dopamine supersensitivity in mesolimbic areas, accompanied by an increase in dopamine receptor density [10]. It must be noted, however, that these neuroadaptations could be secondary to the long-term administration of dopaminergic medications such as DAs and levodopa [10]. Nonetheless, LEDD was not associated with impulsivity measures in the current cohort, indicating that ceruloplasmin may be associated with impulsivity independently of the effects of dopaminergic medications.

## 5. Limitations

A number of limitations of the current study must be noted. Firstly, being a validated measure of the construct of impulsivity, the self-reported nature of the BIS-11 may introduce a degree of bias in the gathered responses due to patients tending to provide what they consider to be more socially desirable responses and being less inclined to admit to impulsive behaviours. In addition, although the absence of a healthy control group for the neuropsychological studies may be considered a limitation, it does not detract from the primary objective and outcome of our study, which was to determine if there was an association between serum ceruloplasmin levels and measures of impulsivity in a carefully studied PD cohort. Finally, elevated ceruloplasmin can be a result of infectious and inflammatory conditions; however, such information was not collected in this retrospective cohort as it was deemed to be outside the scope of the aims. While a limitation, the association between heightened impulsivity and serum ceruloplasmin still remains, regardless of the cause of the elevated serum ceruloplasmin levels.

## 6. Conclusion

To date, this is the first study to explore the relationship between serum ceruloplasmin levels and impulsivity in PwP. Our findings in this PD cohort have shown that participants with high ceruloplasmin levels had higher impulsivity scores as measured on the BIS-11 scale, specifically in relation to nonplanning impulsivity, and not in motor or attentional impulsivity domains. Although females had higher levels of ceruloplasmin and were predominant in the high ceruloplasmin group, the association with impulsivity was independent of gender. The nature of this association remains unclear, but it is proposed that changes in iron and copper homeostasis and dopamine regulation in the basal ganglia and limbic system may play a part. In light of these findings, further investigation is warranted in other PD cohorts using the BIS-11 scale, as well as objective measures of impulsivity and response inhibition.

## Figures and Tables

**Table 1:** Summary of demographic and clinical data in PD patient cohort.

Variable		Mean (SD) or *n*
Gender	Male	135
	Female	79
Patient age (years)		64.44 (9.39)
Disease duration (years)		8.90 (5.93)
Total levodopa (mg/day)		884.93 (616.84)
Dopamine D2 agonist	Yes	99
	No	115
MDS-UPDRS III		19.81 (14.03)
Hoehn and Yahr		1.68 (0.97)
ACE-R		88.55 (10.57)
Mean ceruloplasmin (g/L)		0.26 (0.06)

MDS-UPDRS III, Movement Disorder Society-Unified Parkinson's Disease Rating Scale III; ACE-R, Addenbrooke's Cognitive Examination Revised.

**Table 2:** Univariate analysis of dichotomised ceruloplasmin grouping and clinical measures.

Variable	Normal ceruloplasmin (*N* = 158)	High ceruloplasmin (*N* = 56)	Significance (*p* value; Cohen's *d*)
Mean ceruloplasmin (g/L)	0.23 (0.04)	0.34 (0.05)	*p* < 0.001
Gender (male %)	70.3%	42.9%	*p* < 0.001
Patient age (years)	64.01 (9.44)	65.64 (9.21)	*p*=0.331; *d* = 0.17
Disease duration (years)	8.92 (6.24)	8.83 (5.02)	*p*=0.598; *d* *=* 0.02
Total levodopa (mg/day)	872.62 (598.17)	950.81 (657.16)	*p*=0.482; *d* *=* 0.12
Dopamine agonist (%)	44.3%	51.8%	*p*=0.335
MDS-UPDRS III	18.35 (13.82)	23.91 (13.94)	*p*=0.011; *d* = 0.40
Hoehn and Yahr	1.57 (0.92)	2.04 (0.99)	*p*=0.002; *d* = 0.49
ACE-R	87.72 (11.14)	91.02 (8.23)	*p*=0.034; *d* = 0.34

MDS-UPDRS III, Movement Disorder Society-Unified Parkinson's Disease Rating Scale III; ACE-R, Addenbrooke's Cognitive Examination Revised.

**Table 3:** Generalised linear regression models for total BIS-11 and 2nd order and 1st order outcomes in normal and high ceruloplasmin groups.

Variable	Total (*n* = 214)	Low (*n* = 158)	High (*n* = 56)	Naïve comparison	Corrected comparison
BIS-11 total score	60.89 (0.63)	60.27 (0.77)	62.63 (1.06)	0.109	0.224
2nd order attentional	15.66 (0.24)	15.59 (0.29)	15.86 (0.43)	0.615	0.670
1st order attentional	10.38 (0.19)	10.35 (0.22)	10.45 (0.33)	0.864	0.427
1st order cognitive instability	5.28 (0.11)	5.23 (0.13)	5.41 (0.19)	0.422	0.209
2nd order nonplanning	23.91 (0.35)	23.40 (0.41)	25.34 (0.62)	0.012	0.003
1st order self-control	12.39 (0.23)	12.12 (0.27)	13.14 (0.45)	0.047	0.020
1st order cognitive complexity	11.52 (0.18)	11.28 (0.22)	12.20 (0.34)	0.028	0.009
2nd order motor	21.29 (0.25)	21.28 (0.29)	21.30 (0.49)	0.991	0.921
1st order motor	13.71 (0.20)	13.69 (0.23)	13.75 (0.39)	0.895	0.946
1st order perseverance	7.58 (0.12)	7.59 (0.14)	7.55 (0.23)	0.767	0.898

## Data Availability

The data that support the findings of this study are available from the corresponding author upon reasonable request.
